# Protective Effect of *Pyrus ussuriensis* Maxim. Extract against Ethanol-Induced Gastritis in Rats

**DOI:** 10.3390/antiox10030439

**Published:** 2021-03-12

**Authors:** Naila Boby, Muhammad Aleem Abbas, Eon-Bee Lee, Zi-Eum Im, Walter H. Hsu, Seung-Chun Park

**Affiliations:** 1 Laboratory of Veterinary Pharmacokinetics and Pharmacodynamics, College of Veterinary Medicine, Kyungpook National University, Daegu 41566, Korea; nailaboby@knu.ac.kr (N.B.); syedaleemabbas77@knu.ac.kr (M.A.A.); eonbee@knu.ac.kr (E.-B.L.); 2 Institute of Forest Resources Development, Gyeongsangbuk-do, Andong-si, Gyeongsangbuk-do 36605, Korea; zium78@korea.kr; 3 Department of Biomedical Sciences, College of Veterinary Medicine, Iowa State University, Ames, IA 50011, USA; whsu@iastate.edu

**Keywords:** *Pyrus ussuriensis* Maxim, GC-MS, HPLC analysis, antioxidant, IC_50_, gastritis, ulcer scoring, leucocytes common antigen, prostaglandin E_2_, H^+^/K^+^ ATPase

## Abstract

*Pyrus ussuriensis* Maxim (Korean pear) has been used for hundreds of years as a traditional herbal medicine for asthma, cough, and atopic dermatitis in Korea and China. Although it was originally shown to possess anti-inflammatory, antioxidant, and antiatopic properties, its gastroprotective effects have not been investigated. In the present study, we evaluated the protective effects of *Pyrus ussuriensis* Maxim extract (PUE) against ethanol-induced gastritis in rats. The bioactive compound profile of PUE was determined by gas chromatography mass spectroscopy (GC-MS) and high-performance liquid chromatography (HPLC). The gastroprotection of PUE at different doses (250 and 500 mg/kg body weight) prior to ethanol ingestion was evaluated using an in vivo gastritis rat model. Several endpoints were evaluated, including gastric mucosal lesions, cellular degeneration, intracellular damage, and immunohistochemical localization of leucocyte common antigen. The gastric mucosal injury and ulcer score were determined by evaluating the inflamed gastric mucosa and by histological examination. To identify the mechanisms of gastroprotection by PUE, antisecretory action and plasma prostaglandin E_2_ (PGE_2_), gastric mucosal cyclic adenosine monophosphate (cAMP), and histamine levels were measured. PUE exhibited significant antioxidant effects with IC_50_ values of 56.18 and 22.49 µg/mL for 2,2-diphenyl-1-picrylhydrazyl (DPPH) and 2,2′- azino-di-(3-ethylbenzothiazoline)-6-sulfonic acid (ABTS) inhibition (%), respectively. In addition, GC/MS and HPLC analyses revealed several bioactive compounds of PUE. Pretreatment with PUE significantly (*p* < 0.05) decreased the ulcer index by preventing gastric mucosal lesions, erosion, and cellular degeneration. An immunohistochemical analysis revealed that PUE markedly attenuated leucocyte infiltration in a dose-dependent manner. The enhancement of PGE_2_ levels and attenuation of cAMP levels along with the inhibition of histamine release following PUE pretreatment was associated with the cytoprotective and healing effects of PUE. In contrast, the downregulation of the H^+^/K^+^ ATPase pathway as well as muscarinic receptor (M_3_R) and histamine receptor (H_2_R) inhibition was also involved in the gastroprotective effects of PUE; however, the expression of cholecystokinin-2 receptors (CCK_2_R) was unchanged. Finally, no signs of toxicity were observed following PUE treatment. Based on our results, we conclude that PUE represents an effective therapeutic option to reduce the risk of gastritis and warrants further study.

## 1. Introduction

Ethanol-induced gastritis is a major gastrointestinal (GI) disorder characterized by a condition in which the stomach mucosa becomes inflamed [1,2]. When the stomach is continuously exposed to various substances that have the capacity to cause epithelial damage, it produces less mucus and other substances that normally protect the gastric mucosa from the acidic digestive fluid. Normally mucosal integrity is maintained by defensive factors including an epithelial barrier, blood flow, bicarbonate, mucus secretion, prostaglandins, and growth factors. When noxious factors overwhelm these defensive factors, mucosal injury occurs [3,4]. Long-term alcohol intake can cause acute and chronic gastric mucosal injury [5,6]. This acute and chronic gastritis increases gastric acid secretion. The gastric acid secretion pathways include activation of H_2_R by histamine, activation of M_3_R by acetylcholine, and activation of CCK_2_R by gastrin. H_2_R activation through the cAMP-dependent pathway and M_3_R, and CCK_2_R activation through the Ca^+^-dependent pathway, in turn, activate H^+^/K^+^ ATPase in parietal cells. This enhances the hydrochloric acid concentration in gastric fluids, which leads to gastric mucosal damage [2,7]. Several drugs, such as ranitidine and famotidine (histamine H_2_ receptor blockers) and omeprazole and lansoprazole (proton pump blockers), are used for the treatment of gastritis; however, they may cause adverse effects along with the development of tolerance and relapse [8]. In view of the inadequate therapeutic options available for gastric disorders, there is a need for more effective and safer gastroprotective agents.

Natural products offer a new therapeutic approach, as they represent safe and effective gastroprotective agents [9]. Several studies have evaluated the beneficial effects of extracts from herbal sources for the prevention of gastric injury. Based on these studies, three main activities of natural products are responsible for gastric mucosal protection which include antioxidant, antisecretory, and cytoprotective effects [10]. Pears (*Pyrus* spp.) belong to the Rosaceae family and have significant commercial value. *Pyrus ussuriensis* Maxim (PU) is the major cultivated specie of genus pear, which also includes *Pyrus calleryana* and *Pyrus pyrifolia*, and is primarily distributed in Korea, China, and Japan [11]. Whole fruit and different parts of *Pyrus* spp. are consumed not only as fruit but also for various medicinal purposes, including their antitussive, antioxidant, diuretic, antimicrobial, and anti-inflammatory effects. Pears are also consumed worldwide as a treatment for alcoholic hangover [12,13,14]. Ethanol-induced gastric pathologies are believed to be initiated and aggravated by oxidative stress and reactive oxygen species (ROS). The antioxidant effect prevents such pathologies by reducing oxidative stress and inhibiting free radical production [15]. *Pyrus ussuriensis* Maxim has been reported to have an antioxidant-rich component profile. Several phenolic compounds, such as 4-hydroxyphenyl β-D-glucopyranoside (β-arbutin) and chlorogenic acid, have been identified in pears. Other reported constituents include (−)-epicatechin, (+)-catechin, isorhamnetin, kaempferol, quercetin, and their glycosides. These molecules have been reported to have potential antioxidant effects [16,17,18]. Thus, *Pyrus ussuriensis* Maxim extract may exhibit gastroprotective effects; however, there is no scientific evidence for its gastroprotective effects against gastritis. Therefore, we studied the effect and mechanism of a 50% ethanol extract of *Pyrus ussuriensis* Maxim (PUE) on the repair of gastric mucosa by examining histological and ultrastructural changes and relative gene expression in a rat model. We also evaluated the effects of PUE in relation to the modification of biomarkers including plasma histamine and H_2_-receptors, gastric mucosal cyclic adenosine monophosphate (cAMP), and prostaglandin E_2_ (PGE_2_).

## 2. Materials and Methods

### 2.1. Chemicals/Reagents

Analytical grade chemicals were used in all experiments. For PUE preparation and analysis, ethanol, acetonitrile, and phosphoric acid (H_3_PO_4_) were purchased from the Duksan Company (Ansan, Korea). Monobasic Potassium Phosphate (KH_2_PO_4_), chlorogenic acid, and lansoprazole were purchased from the Sigma Chemical Company (St. Louis, MO, USA). The PGE_2_ ELISA kit was purchased from Abnova (Walnut, CA, USA), whereas cAMP and histamine were measured by a cAMP ELISA kit (Cell Biolabs, San Diego, CA, USA) and histamine assay kit (Abnova, Walnut, CA, USA), respectively.

### 2.2. Plant Material Sampling and Extract Preparation

The dried *Pyrus ussuriensis* Maxim fruit body was ground to a powder, and an extract was prepared using 50% ethanol. Afterwards, the filtered extract was concentrated in a rotary vacuum evaporator (Buchi R-114, BÜCHI Labortechnik AG, Flawil, Switzerland) at 95 °C. The concentrated extract was then dried by using a vacuum freeze drier (Bio Tron, Gangneung, Korea). Brix measurements were performed with a Pocket refractometer PAL-1 (ATAGO, Tokyo, Japan) according to the manufacturer’s instructions to determine the sugar content of PUE.

### 2.3. Phytochemical Profile of PUE

#### 2.3.1. Gas Chromatography Coupled Mass Spectroscopy Analysis

Gas chromatography coupled mass spectroscopy (GC-MS) was used to evaluate the phytochemical profile of PUE and was performed using an Agilent 7890 gas chromatograph (Agilent Technologies, Santa Clara, CA, USA). An Agilent Technologies 5975 mass selective detector (MSD; HP 5973, Hewlett-Packard, CA, USA) was used with an ionization energy of 70 eV and a scanning mass ranging from 10 to 800 m/z. The extract was analyzed with a run time of 67 min using an Agilent J & B DB-5MS column (250 µm internal diameter × 40 m length and 0.25 µm film thickness fused silica capillary column), maintained initially at a temperature of 70 °C for 1 min, followed by 5 °C/min of heating to 300 °C for 20 min. Helium gas was used as a carrier at a flow rate of 1 mL/min with inlet and detector temperatures of 250 °C and a split-less mode injection method.

#### 2.3.2. Identification of Bioactive Compound by HPLC Analysis

An HPLC Agilent Technologies 1100 series (Santa Clara, CA, USA) system consisting of a reverse-phase Eclipse Plus C18 column (particle size, 4.6 mm × 250 mm, 5 µm), a quaternary HPLC pump, an autosampler, and a UV detector was used to analyze PUE. The extract was analyzed for one of its most important bioactive compounds, chlorogenic acid, by HPLC using the method described by Cui. T. et al. with a slight modification [12]. A stock solution of chlorogenic acid at 0.1 mg/mL was prepared in 70% ethanol followed by preparation of different standard concentrations ranging from 0.05 to 10 µg/mL by serial dilution. For HPLC analysis, the analytical conditions consisted of ACN and 0.5% aqueous phosphoric acid (11.5:88.5 ratio, *V*/*V*) as the mobile phase, an injection volume of 20 µL, a 40 °C column temperature, and a wavelength of 327 nm. The PUE solution was then prepared by mixing 20 mg dried extract powder in 70% ethanol followed by 30 min of sonication, vortex mixing, and centrifugation at 1340× *g* for 5 min. The supernatant was removed.

### 2.4. Antioxidant Effects

#### 2.4.1. DPPH Radical Scavenging Activity

The stable free radical, 2,2-diphenyl-1-picryl-hydrazyl (DPPH), was used to evaluate the radical scavenging activity of the antioxidant compounds of PUE. In this assay, ascorbic acid was used as a standard. Different concentrations of PUE (1, 5, 10, 50, 100, and 1000 µg/mL) were added to the 0.1 mM DPPH solution. The absorbance was measured at 517 nm using a spectrophotometer (EPOCH™-2, BioTek instruments, Seoul, Korea) [19]. The radical scavenging activity (RSA) was expressed as a percentage, and IC_50_ values were calculated from the graph in triplicate.

#### 2.4.2. ABTS Radical Scavenging Activity

The 2,2′-azino-bis (3-ethylbenzothiazoline)-6-sulfonic acid (ABTS) radical scavenging activity was determined to evaluate the antioxidant activity of PUE. ABTS contains free radicals in its oxidized form, and it is an electron donor. ABTS ammonium salt (7 mM) and potassium persulfate (2.45 mM) were added to water, and the solution was placed in the dark for 14 h at room temperature where it developed a dark blue color [20]. In each well of a 96-well plate, 200 µL of 7 mM ABTS solution was added followed by the addition of 10 µL/well of the serially diluted PUE. The reaction proceeded for 7 min at RT, and the absorbance was measured at 734 nm. The ABTS and PUE solutions were freshly prepared for each assay. The ABTS scavenging capacity of the PUE was calculated as % of ABTS radical scavenging activity using the following formula: ABTS radical scavenging activity (%) = [1 − (OD_control_ − OD_Sample_/OD_control_)] * 100, where OD_PUE_ is the absorbance of the ABTS radical solution mixed with PUE, and OD_control_ is the absorbance of the ABTS radical solution without PUE. The half-maximal inhibitory concentration of PUE (IC_50_) required for the inhibition of 50% ABTS free radical activity was calculated in triplicate measurements and used to measure the antioxidant potential.

### 2.5. Animal Ethics and Housing

The Animal Care and Use Committee of Kyungpook National University, Daegu, Korea (KNU 2018-107), approved the experimental protocol. All procedures involving the in vivo studies and the experimental protocols were conducted in accordance with the Guide for the Care and Use of Laboratory Animals published by the US National Institute of Health [21]. Fifty 5-week-old male Sprague-Dawley (SD) rats (150–180 g) were obtained from Orient Bio, Inc. (Gyeonggi-do, Korea). The total number of rats used in the study was calculated by the G*power program (3.1.9.2) based on effect size (0.5), α error probability (0.05), power (1-β error probability) (0.8), and group number (5). The animals were acclimatized in a controlled RT (23 ± 3 °C) environment with a relative humidity of 50 ± 10% and a light/dark (12/12) cycle for 7 days before experimentation. The animals were allowed to access sterile water and a standard pellet diet ad libitum.

### 2.6. Experiment Design for In-Vivo Study

**Figure 1:**
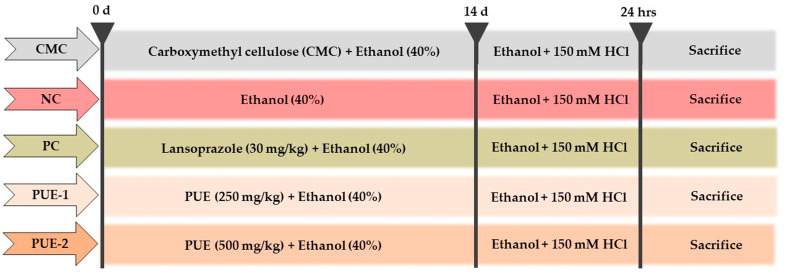
In vivo experimental design for ethanol-induced gastritis in rats. The *Pyrus ussuriensis* Maxim extract (PUE) was administered orally prior to the ingestion of ethanol (40%) for 14 days once a day. Then, 1 h before sacrifice, the rats were administered 70% ethanol in 150 mM HCl. For these experiments, Lansoprazole (30 mg/kg) was used as positive control.

Fifty male SD rats were randomly distributed into five groups (*n* = 10): uninjured (vehicle control group, carboxymethyl cellulose), injured (negative control group), LPZ (positive control group receiving 30 mg/kg b.w of lansoprazole), PUE-250 (low-dose treatment group receiving PUE at 250 mg/kg b.w), and PUE-500 (high-dose treatment group receiving PUE at 500 mg/kg b.w) (Figure 1). The experiment was carried out according to the method described by Arab et al. [22]. The test agents were freshly prepared just before oral administration, and following the administration of the final drug, a single dose of 1 mL of 40% ethanol was administered to the rats in all groups, except the vehicle control group. Each administration was performed based on the most recently recorded body weight for 14 days.

### 2.7. Necropsy and Plasma Sampling

On the fifteenth day, rats from each group received 1 mL of 70% ethanol in 150 mM HCl. At the end of the experiment, the rats were euthanized by carbon dioxide inhalation, and blood samples were collected from the abdominal aorta. Blood was centrifuged (1,600,111× *g*) for 10 min at 4 °C to obtain plasma. The plasma samples were stored at −70 °C prior to biochemical analysis.

### 2.8. Isolation of the Stomach and Quantification of Injury Index for the Gastric Mucosa

Gastritis is characterized by an inflamed gastric mucosa. To determine the extent of inflammation or injury, ulcer scoring or the ulcer index (UI) is frequently used as a standard. The stomachs were opened along the greater curvature, and after washing with phosphate buffered saline (PBS), the stomach was examined for gross findings. Moreover, to determine the severity of gastric mucosal injury, the ulceration index of all rat groups was determined using a graded scaled from 0 to 5 according to the Guth standards [23]. This was based on the severity of vascular congestion, erosion of the gastric mucosa, and lesions (Figure 5A). For ulcer scoring, the cleaned stomach was spread onto a corkboard and the stomach lining was analyzed using an eyepiece. Then, a small portion of the stomach was fixed in 10% formalin for histopathological analysis, whereas the remainder was stored for further biochemical analyses.

### 2.9. Gastric Tissue Histology and Immunohistochemistry

Ten percent-buffered formalin was used for embedding the open stomach after cutting the glandular portion into small pieces. The gastric specimens were processed for histopathological examination and immunohistochemically stained for leukocyte common antigen (LCA, CD45). The images obtained after immunohistochemical analysis were further analyzed with Quantitative Pathology and Bioimage Analysis (Qupath) software (v 0.2.3, Edinburg, UK) to calculate the % intraepithelial lymphocytes (IEL) infiltration in an area of approximately 100 cells.

### 2.10. Biochemical Analysis of Plasma and Gastric Mucosa for Prostaglandin E_2_ (PGE_2_), Histamine and cAMP

For determination of PGE_2_ levels, we followed the manufacturer’s instructions from a PGE_2_ ELISA kit (Abnova, USA Cat. No: KA0326). Briefly, the samples were prepared following the instructions and mixed with 50 µL of assay buffer, blue conjugate, and yellow antibody. The wells were washed following overnight (18–24 h) incubation. Finally, a stop solution was added followed by substrate addition and incubation (1 h). After stopping the development, the results were analyzed at an optical density (OD) of 405 nm. For cAMP measurement, we used the cAMP ELISA kit (Colorimetric, Cat. No: STA-500). The absorbance was measured at 450 nm after the addition of the reagents, washing, incubation (1 h), and adding stop solution. The histamine assay kit (Cat. No: AKR-360, Cell Bio labs, San Diego, CA, USA) was used for measuring histamine content following the manufacturer’s instructions. Briefly, 50 µL of each sample was mixed with 200 µL of Colorimetric Probe Mix followed by incubation for 1 h at 37 °C. The reactions were analyzed at 450 nm using a spectrophotometer (EPOCH™-2, BioTek instruments, Seoul, Korea).

### 2.11. Total RNA Extraction and Quantitative Reverse Transcription Polymerase Chain Reaction (PCR) Analysis

Total RNA was extracted from the mucosal tissue using TRIzol reagent, and cDNA was synthesized using cDNA EcoDry Premix (Takara, Shiga, Japan). Relative expression of mRNA was determined on a CFX96 thermocycler (Bio-Rad). The PCR primer amplification was performed with the following primers: β-actin Sense (S): ATGGATGACGATATCGCTG and Antisense (A): ATGAGGTAGTCTGTCAGGT; H_2_R S: ATGGCATTGAAAGTCACC and A: GACCAAAGAGATGGCAAC; M_3_R S: AAGGCACCAAACGCTCATCT and A: GCAAACCTCTTAGCCAGCGT; CCK_2_R S: GTGAAAATGACAGCGAGAC and A: GGAGGGGGTAGGAGGAT; and H^+^/K^+^ ATPase S: GCTACTGCCCAGGGATG and A: GTAACAGAGCCCCACACAA. Real-time PCR (RT-PCR) was performed using the following cycling conditions: activation of enzyme and initial denaturation at 95 °C for 5 min with 40 cycles of amplification and annealing at 54 (CCK_2_R), 60 (H_2_R, H^+^/K^+^ ATPase and β-actin), and 68 °C (M_3_R).

### 2.12. Statistical Analysis

Duncan’s multiple comparisons tests following one-way analysis of variance (ANOVA) was conducted for statistical comparison using Statistical Analysis Systems (SAS) Software, Version 9.4 (SAS Inc., Cary, NC, USA). The results are expressed as the mean ± SEM of at least three replicates, and P-values less than 0.05 were considered statistically significant. The IC_50_ values were calculated using Prism software (GraphPad Software Inc., San Diego, CA, USA).

## 3. Results

### 3.1. Phytochemical Composition of PUE

**Figure 2:**
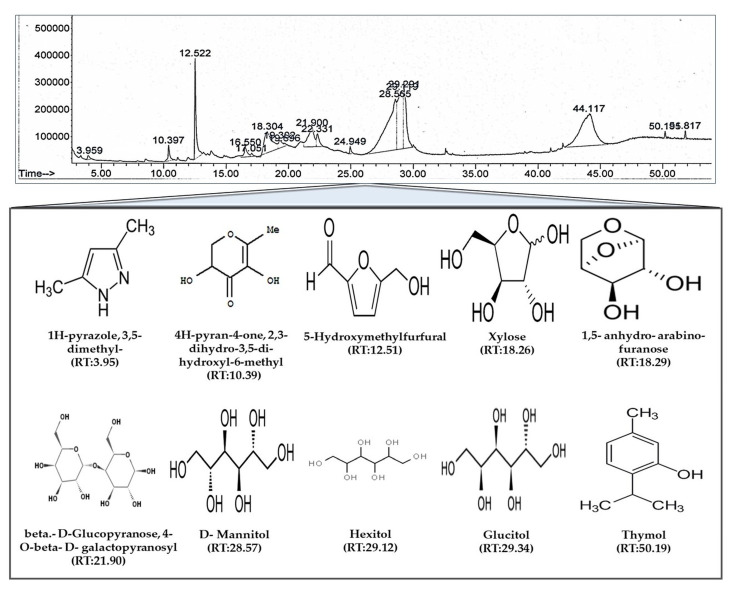
Gas chromatography mass spectroscopy (GC/MS) analysis of *Pyrus ussuriensis* Maxim ethanol (50%) extract. The chromatogram was obtained by GC/MS analysis of PUE. In the chromatogram, each peak represents a specific compound obtained at a unique retention time (RT). The lower part of the figure shows the chemical structures of some of the bioactive compounds along with their RTs.

We used GC-MS analysis to elucidate the bioactive compound profile of PUE and identified 17 compounds, 10 of which have been described previously as exerting antioxidant activity (Figure 2).

**Figure 3:**
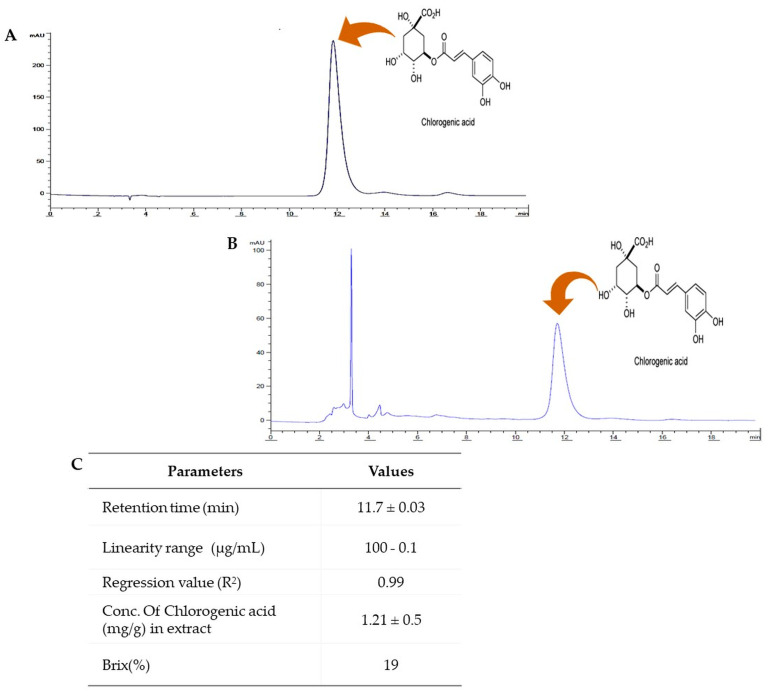
HPLC analysis of *Pyrus ussuriensis* Maxim ethanol (50%) extract. (**A**) The chromatogram obtained by HPLC analysis of the chlorogenic acid standard solution and (**B**) PUE. (**C**) The table shows the different parameters obtained during the method validation procedure and chlorogenic acid content determination using ACN and 0.5% aqueous phosphoric acid (11.5:88.5 ratio, V/V) as the mobile phase, an injection volume of 20 µL, a 40 °C column temperature, and a wavelength of 327 nm.

Extract analysis for carbohydrate content revealed a brix value of 19%. In addition, HPLC was used for quantitative and quality control of the PUE and to measure chlorogenic acid content as an indicative material. The PUE used in the experiment contained a chlorogenic acid content of 1.21 ± 0.5 mg/gm at a retention time of 11.7 ± 0.03 min (Figure 3). Using chlorogenic acid analysis, we further evaluated the antioxidant activity known to be effective at preventing ethanol-induced gastritis as a biological indicator. GC/MS and HPLC analysis exhibited a bioactive compound-enriched profile for PUE.

### 3.2. Antioxidant Activity of PUE

**Figure 4:**
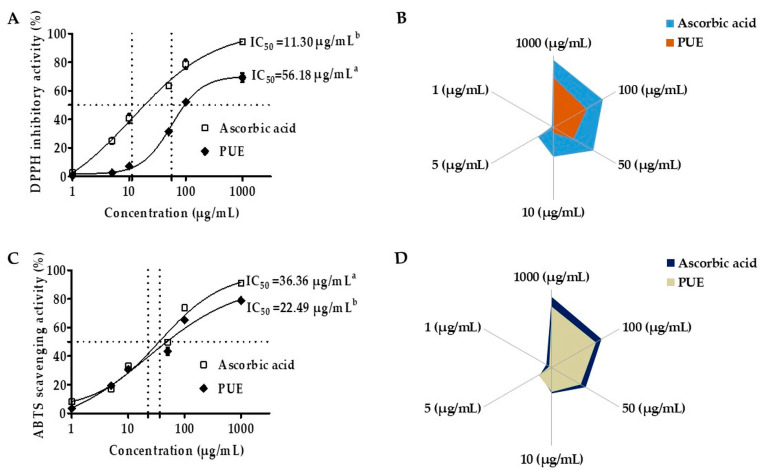
Antioxidant effects of *Pyrus ussuriensis* Maxim ethanol (50%) extract in the 2,2-diphenyl-1-picrylhydrazyl (DPPH) and 2,2′-azino-di-(3-ethylbenzothiazoline)-6-sulfonic acid (ABTS) assays. (**A**) IC_50_ values and (**B**) the % free radical (DPPH) scavenging activity of PUE. The sample and DPPH solutions were incubated at 37 °C and the absorbance was measured at 517 nm. Ascorbic acid was used as a positive control. For the ABTS assay (**C**,**D**), after mixing the ABTS solution and the samples, the absorbance at 734 nm was measured. The results are expressed as the mean % inhibition ± SEM and IC_50_ (*n* = 3).

PUE exhibited concentration-dependent DPPH radical inhibitory activity from 0.1 to 10 mg/mL (Figure 4A,B). The IC_50_ value was 56.18 µg/mL, which was approximately five times higher compared with that of ascorbic acid (11.30 µg/mL). However, the half maximal inhibitory concentration for ABTS was 22.49 µg/mL, which indicated a more potent antioxidant efficacy for PUE compared with ascorbic acid (Figure 4C,D). Our results suggest that PUE is a potent antioxidant agent.

### 3.3. Gross Anatomy of Gastric Mucosa and Ulcer Score in Different Groups

**Figure 5:**
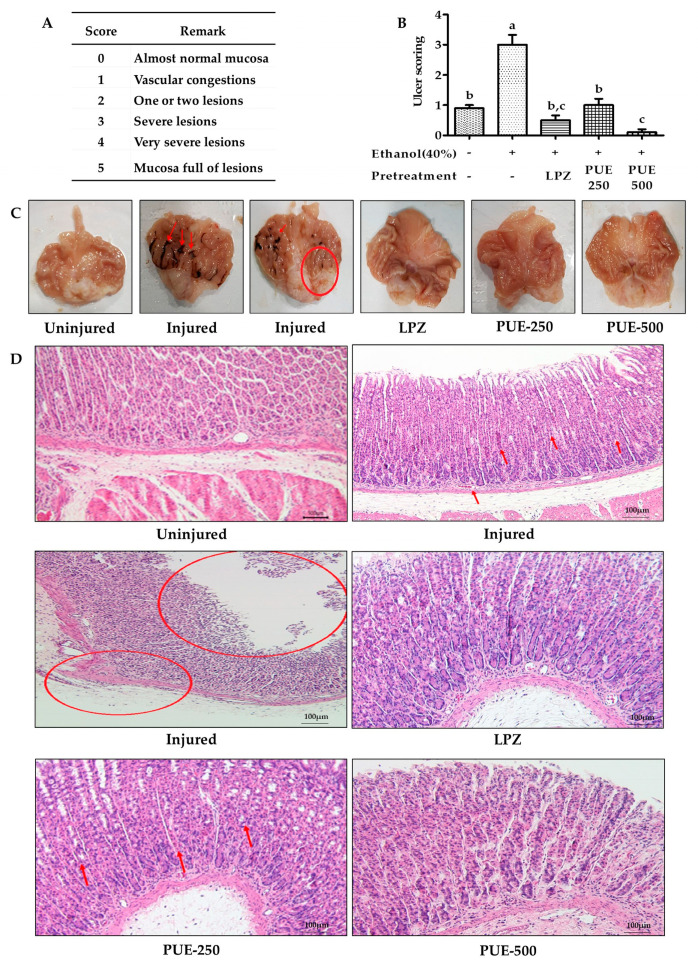
Effects of PUE on ulcer scoring and gastric mucosa histology. (**A**) The Guth standards for gastric ulcer scoring include a physical description of gastric gross anatomy; (**B**) ulcer score variation among different groups of rats, as ethanol induces UI effects in the injured group, was attenuated in PUE (PUE-250 and PUE-500) pretreated groups in a dose-dependent manner; (**C**) gross anatomy of gastric mucosa for different groups of rats including the uninjured group without lesions and injured group with hemorrhagic (as indicated by red arrows) lesions (as indicated by circles); the LPZ (30 mg/kg of lansoprazole) group with smooth mucosal lining, and PUE-250 (250 mg/kg of PUE) and PUE-500 (500 mg/kg of PUE) groups without injury, but with slight congestion of the mucosa; and (**D**) gastric mucosal histology: uninjured group had a smooth gastric mucosal anatomy with normal morphology and color of the parietal cells with distinct cellular structure, glandular pits, and an absence of infiltration; the injured group with cellular degeneration (as indicated by red arrows), mucosal atrophy (as indicated by circles), discoloration of parietal cells depicting the existence of cytoplasmic mucin with hyper-chromatic cells; LPZ, PUE-250, and PUE-500 with mild to moderate degeneration of cells and normal parietal cell coloration (HE staining ×100). The graphical result is presented as means ± SEM for each group and values with different letters indicate significant differences (*p* < 0.05).

The gastric ulcer scoring or ulcer index (UI) is frequently used as a standard to evaluate the extent of gastric mucosal injury. In our study, the mean value of the gastric UI for the treatment groups including lansoprazole (0.5 ± 0.16) and PUE (0.1 ± 0.10) were significantly (*p* < 0.05) lower compared with the injured group (3 ± 0.33) (Figure 5B). The UI results were consistent with those of gross examination, which revealed that almost all of the rats in the injured group developed gastritis as evidenced by gross symptoms, including ruptured membranes, lesions, congestion, and hemorrhagic wounds. While rats receiving lansoprazole or PUE did not show any lesions of the stomach lining, a reduced flattening of the stomach wall was observed in these groups (Figure 5C). Our results suggest that the PUE conferred cytoprotection to the gastric mucosa and could prevent the development of hemorrhagic lesions.

### 3.4. Histological Studies and Immunohistochemistry

The histopathological evaluation revealed that the gastric mucosal appearance of the uninjured group was normal with distinct cellular structures and clear gastric pits. The parietal cells showed a normal morphological color without cell infiltration. Rats from the injured group, however, exhibited highly degenerative gastric mucosal cells and hemorrhage. Furthermore, reductions in cytoplasmic mucin, mucosal atrophy, and hyperchromatic cells were seen in some structures of this group (shown by the circles and arrow). The rats pretreated with lansoprazole and PUE showed protection of the gastric mucosa with mild to moderate cellular degeneration and inflammation (Figure 5D).

**Figure 6:**
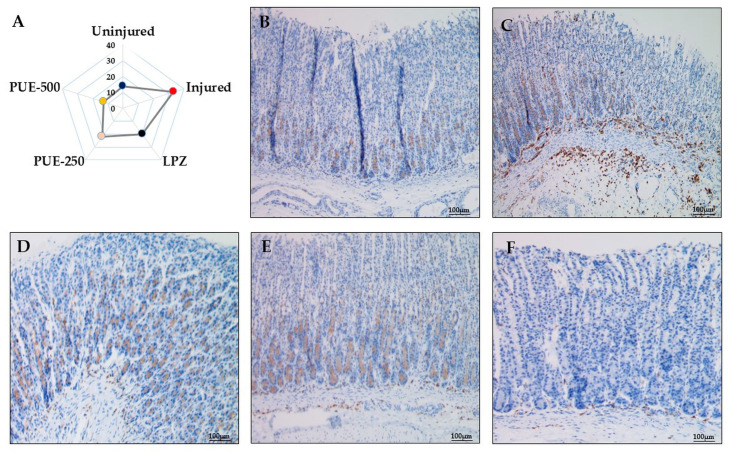
Immunohistochemical analysis of leukocyte common antigen (LCA, CD45) in the treatment groups. (**A**) Radar graph indicating the % IEL infiltration in a selected area of 100 cells; (**B**) in the uninjured group, smooth gastric mucosa showed a normal morphology and presence of a lower number of intraepithelial lymphocytes (fewer brown stains) with no infiltration (×100); (**C**) the injured group showed gastric mucosa with a large number of intraepithelial lymphocytes (more browning) and more infiltration (×100); (**D**) LPZ (30 mg/kg of lansoprazole) showed little infiltration of lymphocytes into the gastric epithelial layer (×100), (**E**) PUE-250 (250 mg/kg of PUE), and (**F**) PUE-500 (500 mg/kg of PUE) groups with mild intraepithelial leukocytes (×100).

Immunohistochemical evaluation of gastric mucosal leukocyte common antigen (LCA, CD45) showed enhanced intraepithelial lymphocyte (IEL) infiltration in the injured group which contained 33% IEL infiltration. However, IEL infiltration was attenuated by pretreatment with lansoprazole with a value of 20% IEL infiltration and treatment with PUE (250 and 500 mg/kg) with IEL infiltration values of 22 and 13%, respectively. A lower number of leucocytes (fewer brown stains) were identified in the submucosal layer of the gastric wall of rats from the treatment groups (Figure 6 and Figures S1–S5). Thus, PUE induced gastroprotection by preventing cellular degeneration and infiltration.

### 3.5. The Changes in PGE_2_, cAMP, and Histamine Levels

**Figure 7:**
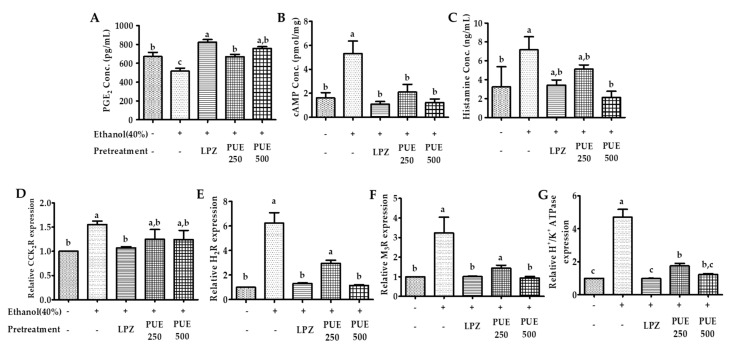
Effects of PUE on plasma biomarkers and relative gene expression. (**A**) Plasma prostaglandin E2 (PGE2) content (pg/mL); (**B**) gastric cAMP (nmol/mL); (**C**) plasma histamine concentration (ng/mL); (**D**–**G**) show the relative gene expression of the CCK receptor, H2-receptor, M3-receptor, and H+/K+ ATPase, respectively. In the graph, all values are expressed as the mean ± SEM, and values with different letters indicate significant differences (*p* < 0.05).

Biomolecular studies of the effect of low and high doses of PUE revealed that compared with the injured group, the plasma PGE_2_ concentrations were significantly (*p* < 0.05) increased in the groups pretreated with lansoprazole and PUE, irrespective of the dose. The plasma histamine assay and gastric mucosal cAMP ELISA showed a significant (*p* < 0.05) decrease in the concentration of histamine and cAMP in the lansoprazole and PUE-treated groups compared with the injured group (Figure 7A−C and Table S1). These results indicate that PUE induced gastroprotection by PGE_2_ enhancement and attenuation of histamine and cAMP levels within the gastric mucosa.

### 3.6. Quantitative Reverse Transcription (qRT) PCR Analysis

In order to determine the gastroprotective effects of PUE in relation to receptor activation or attenuation, the relative expression of the receptor-associated genes was measured by qRT-PCR. The results showed that the expression of M_3_- and H_2_-receptors and H^+^/K^+^ ATPase was significantly (*p* < 0.05) increased following intragastric ethanol administration. However, pretreatment with PUE significantly (*p* < 0.05) downregulated M_3_R, H_2_R, and H^+^/K^+^ ATPase expression (Figure 7E–G and Table S2). However, the results were different for CCK_2_R gene expression. Unlike lansoprazole, pretreatment with PUE had no significant (*p* < 0.05) effects on the expression of the CCK_2_R gene (Figure 7D and Table S2).

## 4. Discussion

### 4.1. Phytochemical Composition

Recently, *Pyrus ussuriensis* Maxim has gained attention because of its high content of therapeutically active components including flavonoids, glycosides, anthocyanins, and total phenolic compounds [16,17,18]. Our results indicated that the major constituent of the 17 compounds present in the extract was 3,5-dimethyl-1H-pyrazole (0.42%), which has been reported to exert similar effects as NSAIDs with antiepileptic, anti-inflammatory, and antimicrobial activities [16]; 4H-pyran-2,3-dihydro-3,5-dihydroxy-6-methyl (2.46%), which is reported to have antiproliferative, anti-inflammatory, antifungal, antioxidant, and pro-apoptotic effects [24,25]; 5-hydroxymethylfurfural (13%), which is reported to have antiproliferative, cardioprotective, hepatoprotective, and antioxidant activities [26,27,28,29]; 1,5-anhydro-arabino-furanose (7.76%), reported to have antioxidant and anti-inflammatory activities [30]; beta-D-Glucopyranose, 4-O-beta-D-galactopyranosyl (5.72%), which is reported to have antioxidant activity [31,32]; and Thymol (0.31%), which has been reported to exhibit anesthetic, synergistic antibacterial, anti-infective, and antifungal activities [33,34,35]. We also identified Hexitol (35.31%) and D-Mannitol (52.95%), mostly used as synonyms, which are well known osmotic diuretic, sweetening, and cathartic agents. Mannitol is also used as the primary drug to treat acute glaucoma in veterinary sciences and has medical application in tissue rejection and as an antioxidant agent [36]. Using HPLC analysis, we also identified CGA, which was found to be the most abundant polyphenol and has therapeutic application because of its broad spectrum of biological properties, including antimicrobial, antioxidant, antiobesity, and anticarcinogenic activities [37,38,39,40]. It is also known to have excellent oral absorption. Moreover, in our extract, the concentration of chlorogenic acid was 1.21 ± 0.5 mg/gm of extract, which is almost higher compared with other published reports [14]. Therefore, because of the abundance of identified phytochemicals and their antioxidant and anti-inflammatory activities, our extract exhibits gastroprotective efficacy.

### 4.2. Antioxidant Effects of PUE

Ethanol-induced gastric damage may be mediated by free radical generation [15]. Hydroperoxy and superoxide anion free radicals are released into the body during ethanol metabolism. A recent study showed that compounds or extracts with significant antioxidant activity could increase gastroprotection and healing effects by the gastric mucus glycoprotein mechanism [41]. Free radicals and ROS are important to ethanol-induced and nonsteroidal anti-inflammatory drug (NSAIDs)-induced mucosal damage [42]. Antioxidants can heal or prevent gastritis by scavenging ROS. Moreover, free radicals such as DPPH and ABTS exhibit scavenging activity and have been used to quantify the antioxidant activity of plant extracts. If the IC_50_ value of an extract is lower, that compound or extract is considered to have a stronger antioxidant activity [20]. According to our results, the IC_50_ values of our extract were 56.18 and 22.49 µg/mL for the inhibitory effects of DPPH and ABTS, respectively, which is far less compared with the IC_50_ value reported previously by other cultivars of *Pyrus ussuriensis* Maxim [11,31,43,44]. We found significant free radical scavenging activity resulting from DPPH, suggesting that the *Pyrus ussuriensis* Maxim extract has significant antioxidant activity, which was subsequently confirmed by ABTS scavenging activity. Thus, the gastroprotective and antigastritis effects of PUE may result from its antioxidant efficacy.

### 4.3. Gastroprotective Effects of PUE

As a folk remedy, *Pyrus ussuriensis* Maxim is known to be effective for treating gastric ulcer and constipation. In the ancient traditional medicine book, “Dongui Bogam”, it is also a part of traditional medicine. Many studies have reported the effects of PU on inflammation, lung disease, cough, asthma, and constipation [14,16,43,44]; however, the effects and mechanisms for treating gastroenteritic conditions such as gastritis have not been studied. Therefore, in this study, we investigated the gastroprotective efficacy of PUE on an ethanol-induced gastritis rat model. Ethanol treatment resulted in extensive gastric mucosal injury in the rats, and pretreatment with PUE conferred protection from ethanol-induced gastric mucosal damage through various mechanisms. Ethanol-induced gastric damage and acute gastric lesions characterized by various pathological alterations including hemorrhage, inflammatory cell infiltration, edema, and loss of epithelial cells are the most frequently used models for studying the pathogenesis of acute gastric injury [45,46]. In our study, the administration of 40% ethanol to rats caused severe hemorrhagic lesions along with an increased ulcer score. These alterations in the gross findings correlated well with extensive histopathological changes, such as vascular congestion, edema, degeneration of epithelial cells, intracellular infiltration, and increased apoptosis of gastric epithelia. Our findings are similar to those of previous reports describing the gastro-protective effects of other plants [47,48,49,50]. Moreover, PUE treatment before ethanol administration tempered gastritis-associated changes within the gastric mucosa, as evidenced by a decrease in the gastric ulcer score, flattening of the mucosal fold in the gastric wall, and leucocyte infiltration. The gastric barrier damage was more severe in the injured group compared with the uninjured and treatment groups based on gross inspection as well as microscopic analysis.

**Figure 8:**
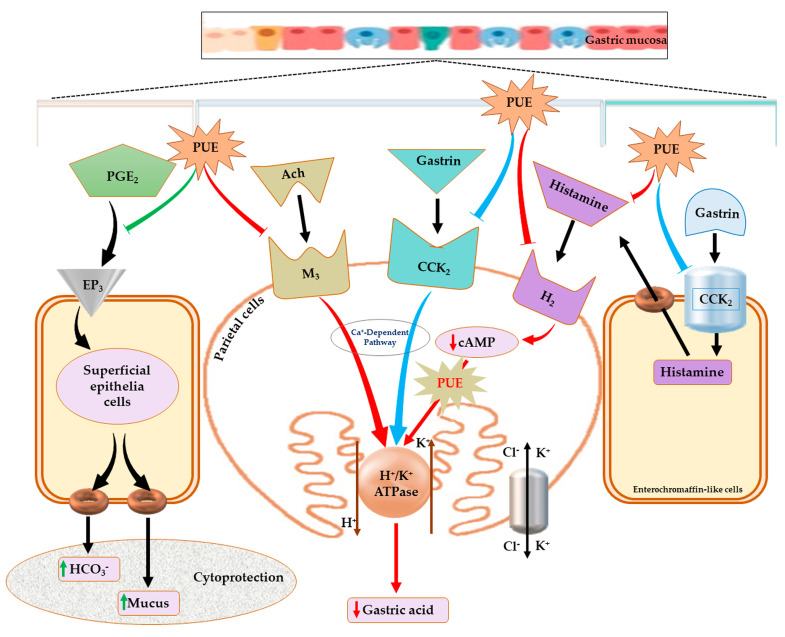
Proposed mechanism for the effects of PUE against gastritis. The schematic pathway illustration indicates that PUE exerts its therapeutic effects against gastritis either by improving cytoprotection through the increased secretion of mucus and bicarbonates following stimulation (green arrow at left side) of PGE_2_ production or healing the inflamed gastric mucosa by attenuating gastric acid secretion (red arrows) through downregulation (red arrows) of H^+^/K^+^ ATPase, H_2_- and M_3_-receptors, and a reduction in cAMP levels. The effects of PUE on the CCK_2_ receptor are not significant (blue arrows), but PUE can possibly decrease histamine concentration by an unknown mechanism.

The gastric mucosal layer is the first line of defense for the stomach against external stimuli, and its motility can affect the development of gastric lesions [51]. Contraction of its circular muscles increases the flattening of the gastric mucosal folds. This results in an increase in the exposed mucosal area for necrotizing agents with an enhanced volume of gastric irritants. Because ethanol intake by rats can induce contraction of the circular muscles of the gastric fundus, damage to the mucosal barrier can lead to the invasion and inflammation of the gastric mucosa [52,53]. Recent studies have shown that damage to the gastric mucosal barrier involves impairment of microcirculation that can lead to the elevation and attenuation of some biochemical parameters involved in maintaining the GI system, such as ET-1 (endothelian-1), NO (nitrous oxide), and PGE_2_. PGE_2_ is a vasodilator factor that can inhibit platelet aggregation and thrombosis, accelerate the flow of the gastric mucosal microcirculation, promote the secretion of bicarbonate and mucus, mediate adaptive immune protective functioning, increase protein synthesis and cell regeneration, and enhance the repair of the damaged gastric mucosa and first line defensive factors [54,55]. Gastric acid is another key factor for normal upper gastrointestinal function involving iron and calcium absorption, protection against bacterial infection, and protein digestion. However, an abnormal level of gastric acid can trigger different pathologies including gastritis [56,57]. The induction of H^+^/K^+^ ATPase by increased intracellular cAMP content and stimulation of the Ca^+^-dependent pathway is involved in the pathogenesis of ethanol-induced gastritis. This has been attributed to the upregulation of receptors (H_2_R, M_3_R, and CCK_2_R) present in the parietal cells of the gastric mucosa [2,7,58]. In our study, the intracellular cAMP and plasma histamine concentration following PUE pretreatment was attenuated compared with the injured group. In addition, H_2_R, M_3_R, and H^+^/K^+^ ATPase gene expression was increased in the injured group, an effect which was antagonized by PUE pretreatment. These results suggest that PUE inhibits gastric mucosal acid secretion through the downregulation of H+/K+ ATPase. This may occur through attenuation of intracellular cAMP levels via the downregulation of H_2_R or by the downregulation of M_3_R. The effects of PUE on CCK_2_R was not significant and different compared with lansoprazole treatment. This finding was in good agreement with that of previous gastroprotection studies [2] and suggests that gastroprotection induced by PUE is associated with the expression of H_2_R, M_3_R, and H^+^/K^+^ ATPase, but not with the expression of CCK_2_R mRNA. Another possibility is related to the cytoprotective effects. We found that PUE augmented the level of PGE_2_ and the mucosal folds that participate in mucus and bicarbonate secretion. Further study is warranted to elucidate the biochemistry of the proposed pathway for PUE-induced gastroprotection, as shown in Figure 8.

## 5. Conclusions

Our study of *Pyrus ussuriensis Maxim* extract revealed an effective phytonutrient profile that may be associated with its antioxidant and antigastritis effects, including enhancing primary and secondary gastric mucosal integrity by increasing mucus and bicarbonate secretion through the prostaglandin (PGE_2_) pathway within the superficial epithelial cells and attenuation of cAMP levels to reduce acid secretion from parietal cells (Figure 8). Furthermore, the beneficial PUE-induced effects are substantiated by reversing the affected expression level of some genes implicated in ethanol-induced gastritis pathologies. Our results suggest that the protective effects of PUE against ethanol-induced gastritis are attributed to the downregulation of H^+^/K^+^ ATPase and the histaminergic and muscarinic receptors within the parietal cells. Therefore, because of its gastro-protective effects, *Pyrus Ussuriensis* Maxim may be used as a potential natural phytomedicine for the treatment of ethanol-induced gastritis. Moreover, this study may be used as a basis for other GI-related effects that may be ameliorated by PUE.

## Data Availability

The data supporting the conclusions of this study are available from the corresponding author upon request.

## Supplementary Materials

The following are available online at https://www.mdpi.com/2076-3921/10/3/439/s1, Table S1: Effects of PUE on plasma prostaglandin E_2_ (PGE_2_) content (pg/mL), gastric cAMP (nmol/mL), and plasma histamine concentration (ng/mL). Table S2: Effects of PUE on the relative gene expression of the CCK_2_ receptor (CCK_2_R), H_2_-receptor (H_2_R), M_3_-receptor (M_3_R), and H^+^/K^+^ ATPase. Figure S1: Immunohistochemical analysis of leukocyte common antigen (LCA, CD45) with IEL count (used for % IEL infiltration) in selected areas for the uninjured group (×100). Figure S2: Immunohistochemical analysis of leukocyte common antigen (LCA, CD45) with IEL count (used for % IEL infiltration) in selected area for the injured groups (×100). Figure S3: Immunohistochemical analysis of leukocyte common antigen (LCA, CD45) with IEL count (used for % IEL infiltration) in selected areas of the lansoprazole-treated groups (×100). Figure S4: Immunohistochemical analysis of leukocyte common antigen (LCA, CD45) with IEL count (used for % IEL infiltration) in selected areas of the group pretreated with 250 mg/Kg of PUE. Figure S5: Immunohistochemical analysis of leukocyte common antigen (LCA, CD45) with IEL count (used for % IEL infiltration) in selected areas of the group pretreated with 500 mg/Kg of PUE.
