# Supplementary material for: Protective Effect of Pyrus ussuriensis Maxim. Extract against Ethanol-Induced Gastritis in Rats
Source: Antioxidants (Basel). 2021 Mar 12;10(3):439. doi: 10.3390/antiox10030439 (PMC8002011; doi:10.3390/antiox10030439)
Supplement: Supplementary file 1 [file antioxidants-10-00439-s001.zip › Supplementary files/Supplementary Tables.docx]

**Supplementary Data**

Table S1. Effects of PUE on plasma prostaglandin E_2_ (PGE_2_) content (pg/mL), gastric cAMP (nmol/mL), and plasma histamine concentration (ng/mL).

| Parameters | Groups | | | | |
| --- | --- | --- | --- | --- | --- |
|  | Uninjured | Injured | Lansoprazole | PUE (250 mg/Kg) | PUE (500 mg/Kg) |
| PGE_2_ Conc. (pg/ mL) | 670.9 ± 45^b^ | 515.8 ± 31.51^c^ | 824.0 ± 27.55^a^ | 670.3 ± 24.88^b^ | 756.9 ± 18.77^a,b^ |
| cAMP Conc. (pmol/ mg) | 1.62 ± 1.2^b^ | 5.33 ± 2.91^a^ | 1.10 ± 0.59^b^ | 2.11 ± 1.80^b^ | 1.23 ± 0.83^b^ |
| Histamine Conc. (ng/ mL) | 3.25 ± 2.1^b^ | 7.17 ± 1.39^a^ | 3.42 ± 0.56^a,b^ | 5.12 ± 0.46^a,b^ | 2.13 ± 0.65^b^ |

In the graph, all values are expressed as the mean ± SEM and values with different letters indicate significant differences (P < 0.05).

Table S2. Effects of PUE on the relative gene expression for the CCK_2_ receptor (CCK_2_R), H_2_-receptor (H_2_R), M_3_-receptor (M_3_R), and H^+^/K^+^ ATPase.

| Gene | Groups | | | | |
| --- | --- | --- | --- | --- | --- |
|  | Uninjured | Injured | Lansoprazole | PUE (250 mg/Kg) | PUE (500 mg/Kg) |
| CCK_2_R | 1.0 ± 0.0^b^ | 1.55 ± 0.07^a^ | 1.072 ± 0.02^b^ | 1.25 ± 0.20^a,b^ | 1.24 ± 0.19^a,b^ |
| H_2_R | 1.0 ± 0.0^b^ | 6.23 ± 0.85^a^ | 1.31 ± 0.04^b^ | 2.95 ± 0.25^a^ | 1.15 ± 0.06^b^ |
| M_3_R | 1.0 ± 0.0^b^ | 3.24 ± 0.80^a^ | 1.01 ± 0.02^b^ | 1.44 ± 0.0.16^a^ | 0.94 ± 0.07^b^ |
| H^+^/K^+^ ATPase | 1.0 ± 0.0^c^ | 4.70 ± 0.47^a^ | 0.98 ± 0.03^c^ | 1.75 ± 0.16^b^ | 1.22 ± 0.06^b,c^ |

In the graph, all values are expressed as the mean ± SEM and values with different letters indicate significant differences (P < 0.05).
