# Supplementary figures and images for: Protective Effect of Pyrus ussuriensis Maxim. Extract against Ethanol-Induced Gastritis in Rats
Source: Antioxidants (Basel). 2021 Mar 12;10(3):439. doi: 10.3390/antiox10030439 (PMC8002011; doi:10.3390/antiox10030439)

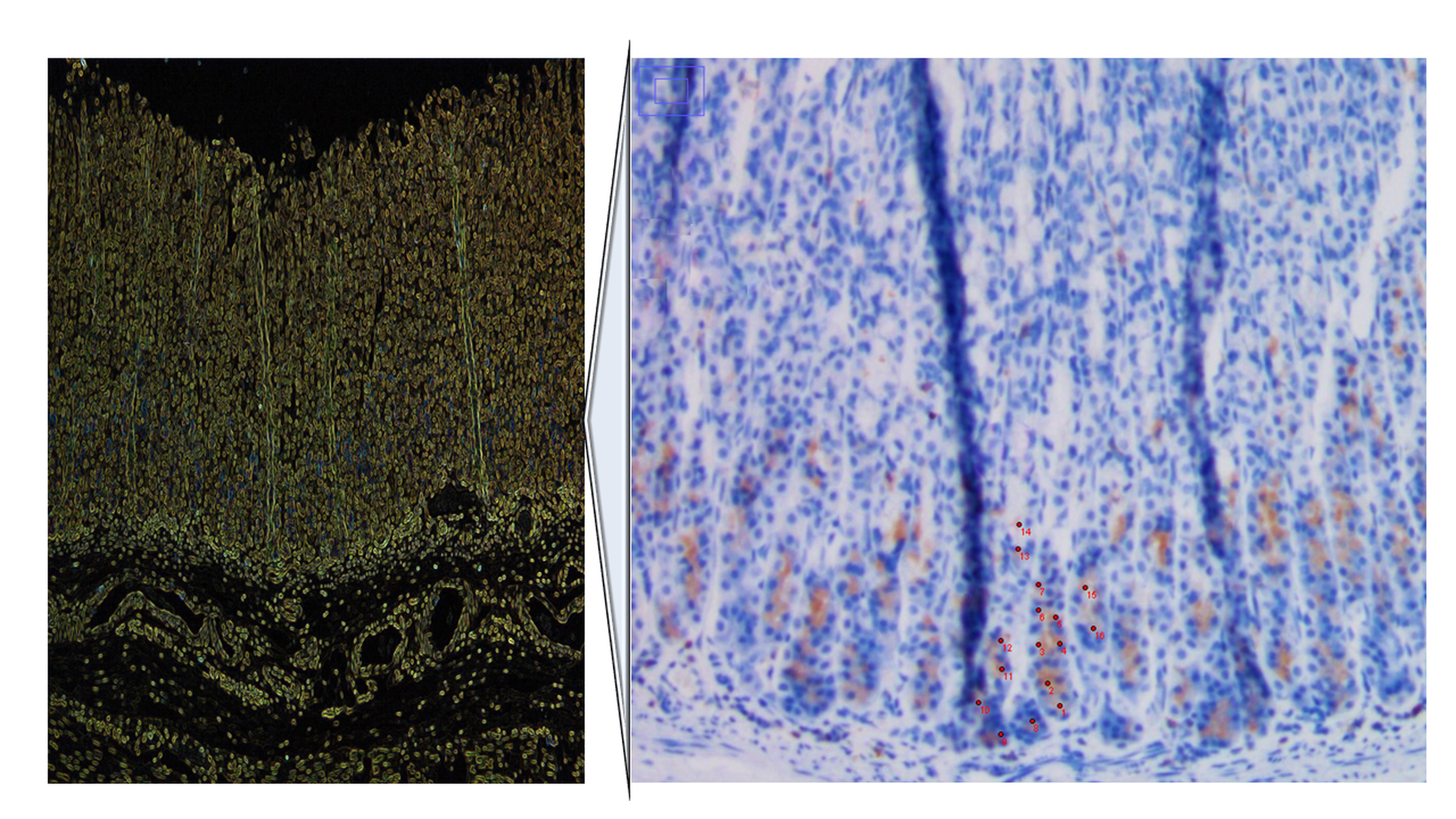

Supplement: Supplementary file 1 [file antioxidants-10-00439-s001.zip › Supplementary files/Supplementary figures/Figure S1.TIF]

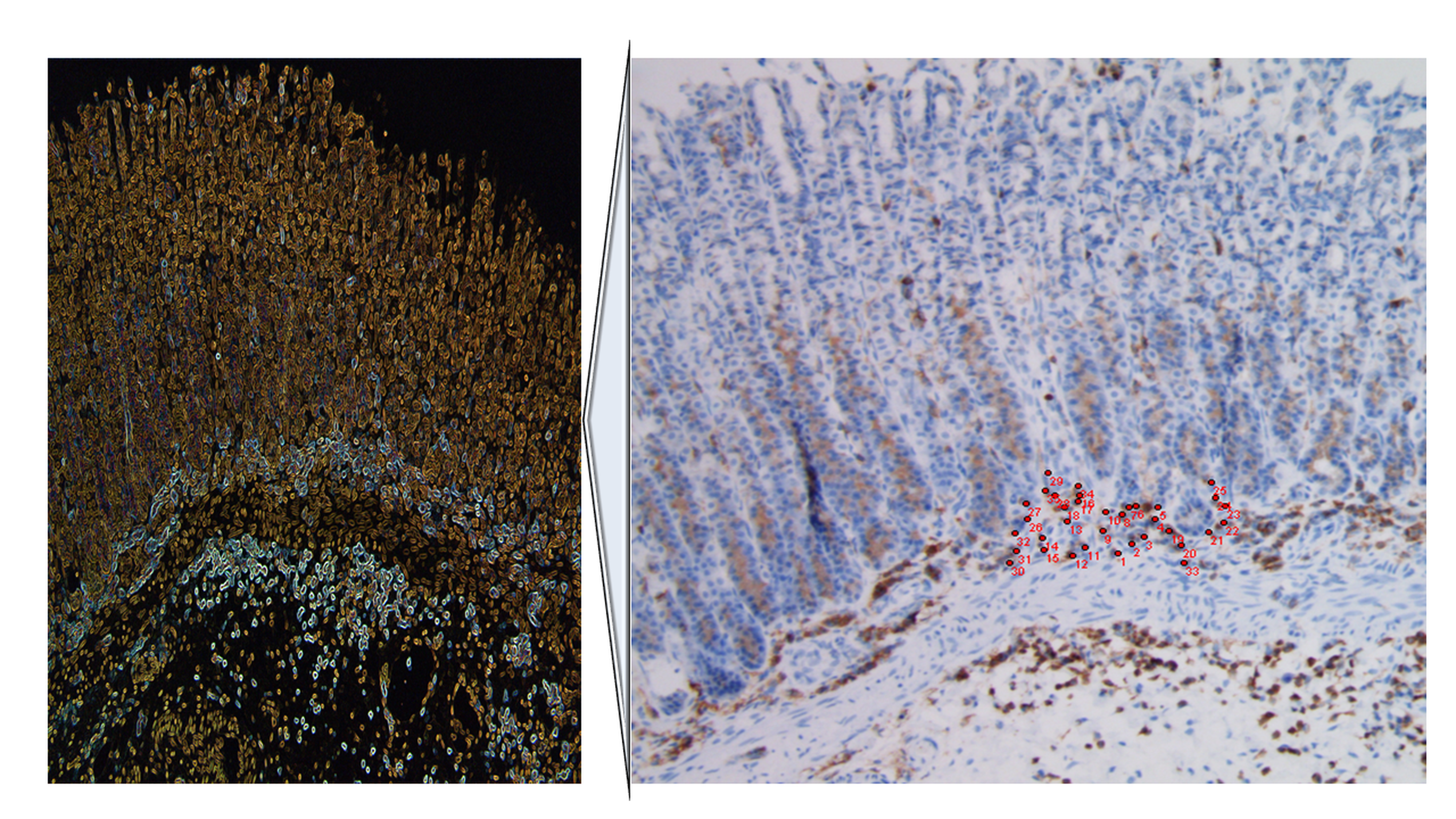

Supplement: Supplementary file 1 [file antioxidants-10-00439-s001.zip › Supplementary files/Supplementary figures/Figure S2.TIF]

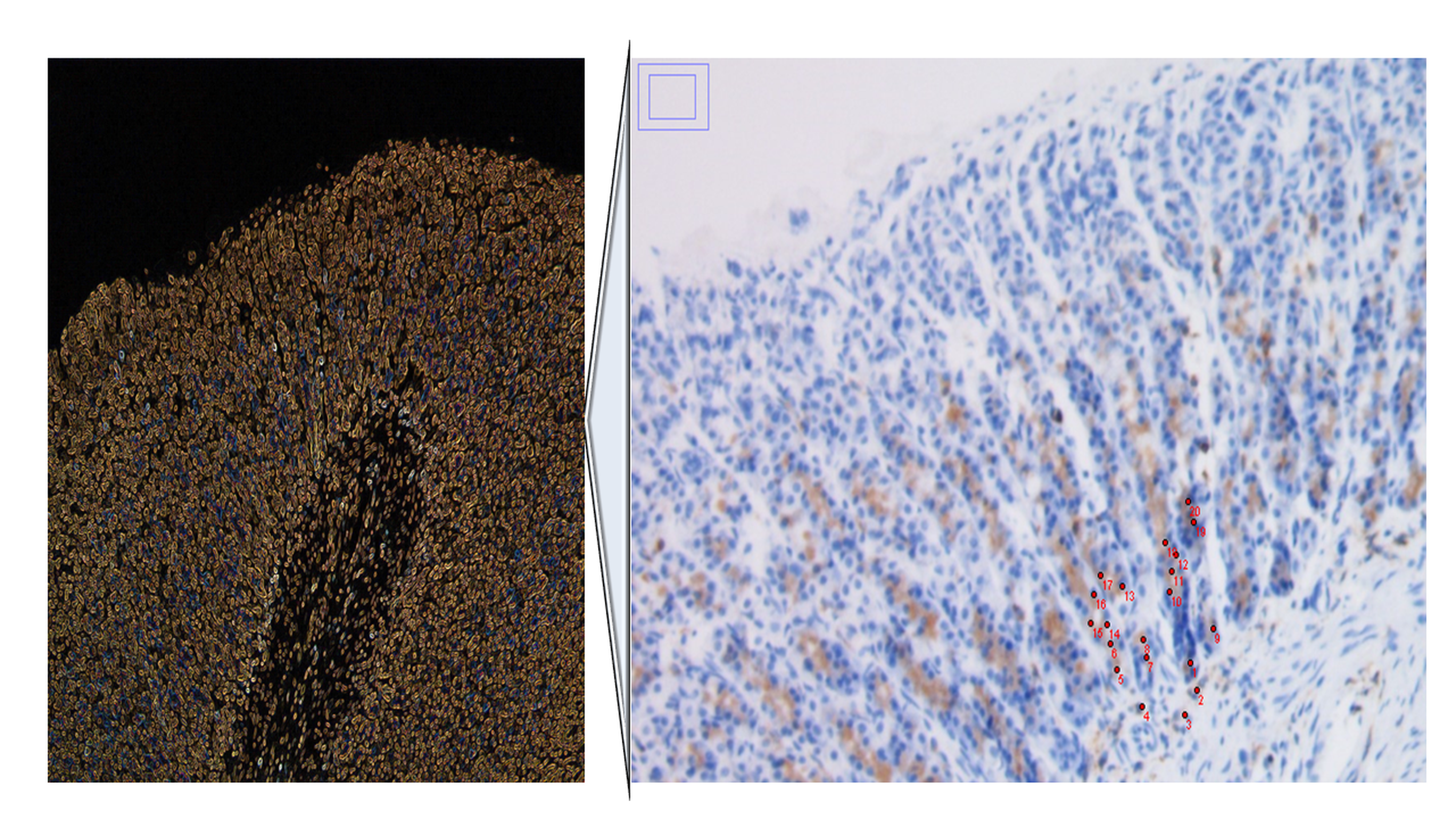

Supplement: Supplementary file 1 [file antioxidants-10-00439-s001.zip › Supplementary files/Supplementary figures/Figure S3.TIF]

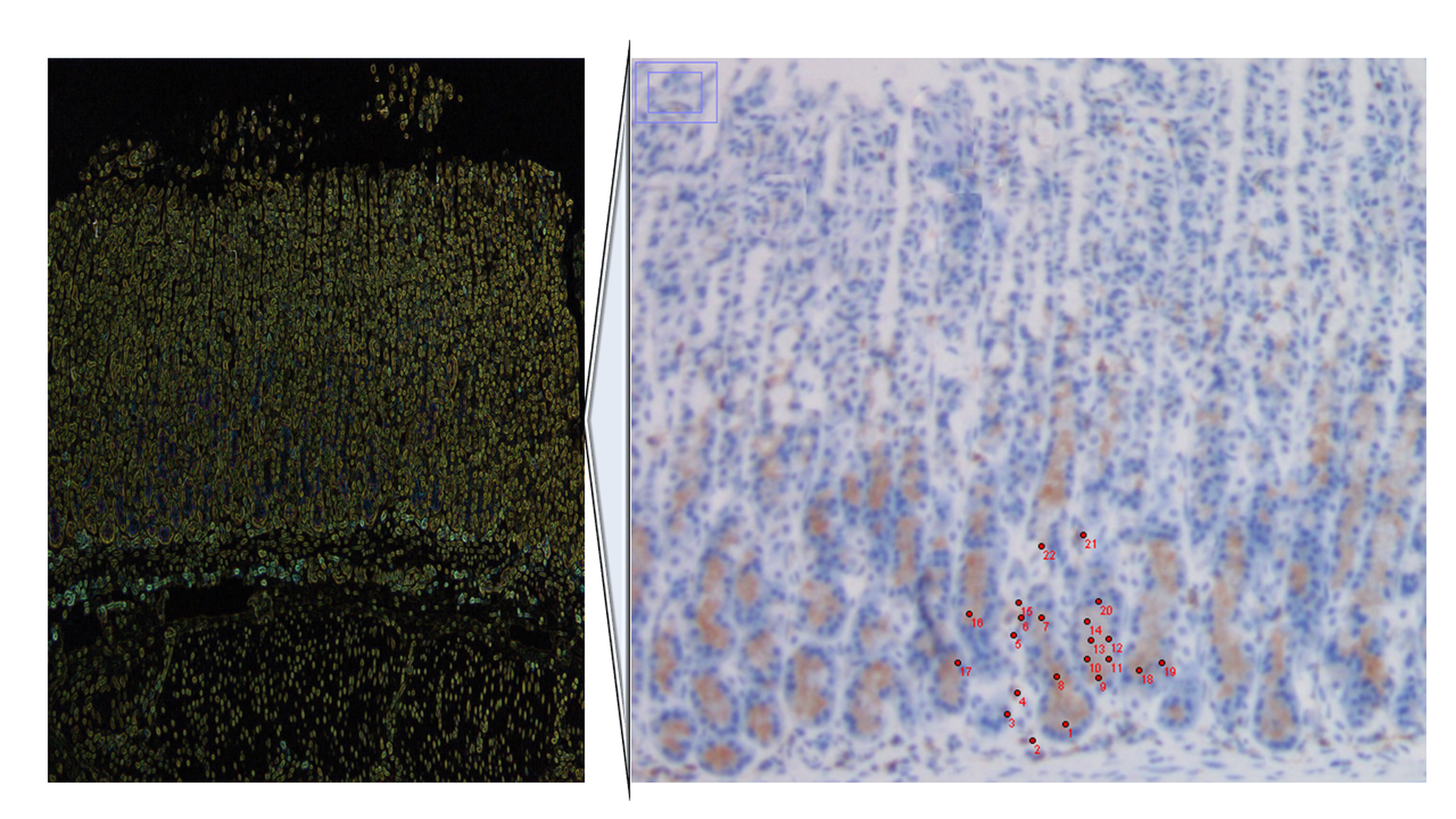

Supplement: Supplementary file 1 [file antioxidants-10-00439-s001.zip › Supplementary files/Supplementary figures/Figure S4.TIF]

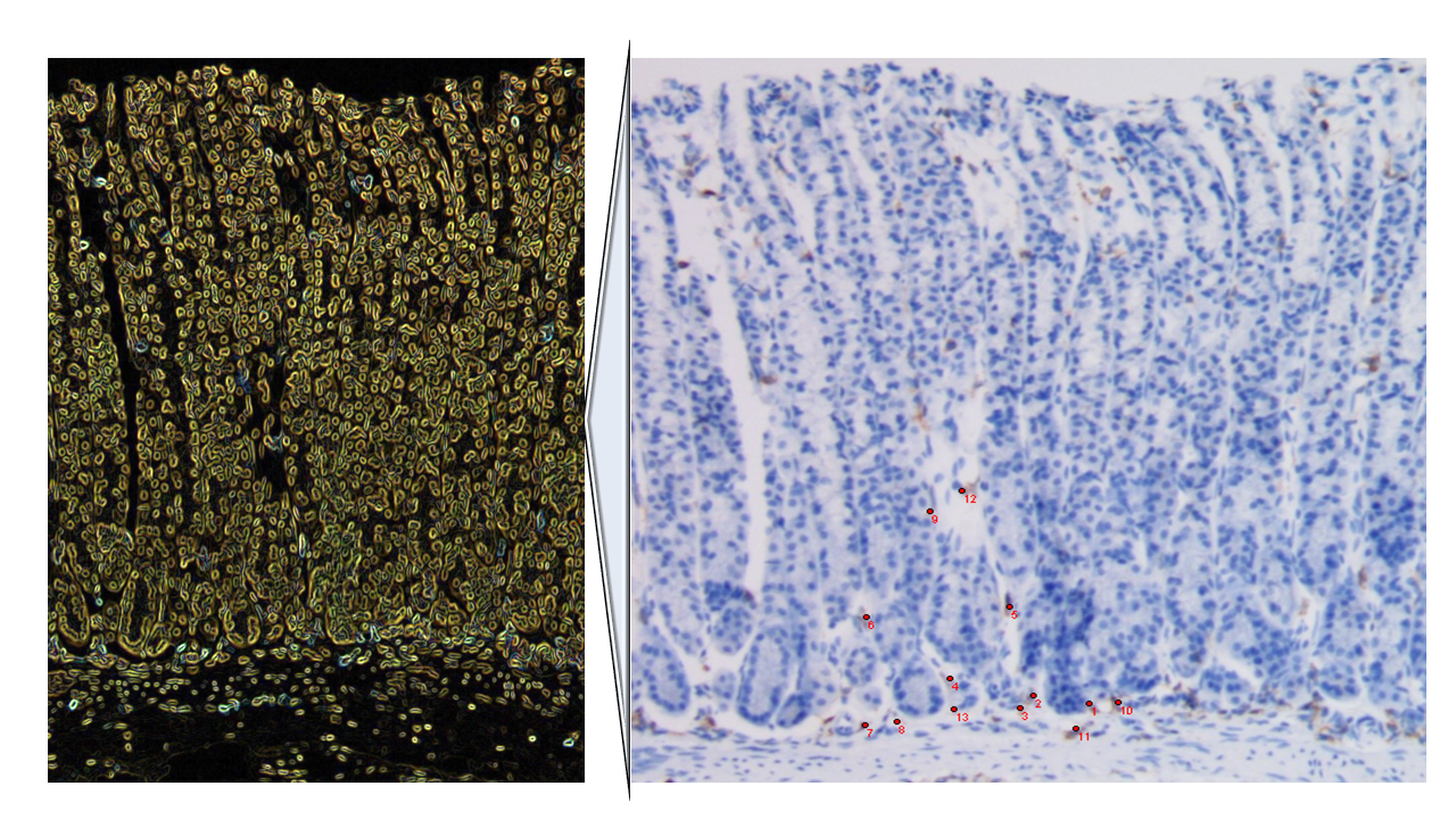

Supplement: Supplementary file 1 [file antioxidants-10-00439-s001.zip › Supplementary files/Supplementary figures/Figure S5.TIF]
